# Dyadic Patterns of Marital Quality During Later Life

**DOI:** 10.1093/geroni/igaa057.1106

**Published:** 2020-12-16

**Authors:** Jennifer Bulanda, Taka Yamashita, J Scott Brown

**Affiliations:** 1 Miami University, Oxford, Ohio, United States; 2 University of Maryland, Baltimore, Maryland, United States

## Abstract

Although earlier cross-sectional studies suggested a U-shaped curve in marital quality over the life course, recent longitudinal studies find stability or continual decline (Proulx, Ermer, & Kanter, 2017). It is important to better understand patterns of marital quality during later life, as marital quality is associated with older adults’ marital stability, health, and longevity. However, few studies have utilized couple-level data to examine marital quality trajectories, and only one has examined dyadic patterns during later life (Wickrama et al., 2020). We use nationally-representative data from the 2006-2016 waves of the Health and Retirement Study (HRS) to examine positive and negative dimensions of marital quality among a sample of continuously-married couples over age 50 in which both partners provided data on marital quality at three time points (n = 1,389 couples). A survey-weighted latent growth curve model simultaneously examines two marital quality trajectories: own and spouse’s. Preliminary results show that mean baseline positive and negative marital quality are similar for own and spouse’s trajectories. Although growth rates are statistically non-significant for positive marital quality, the variance of growth rate is statistically significant for spouse’s trajectory (0.001, p < 0.05), and greater baseline own positive marital quality is associated with negative growth of spouse’s positive marital quality. Growth rates are similar for own and spouse’s trajectories of negative marital quality, and variance of growth rate is statistically significant for spouse’s negative marital quality trajectory. Results point to stability in marital quality over time, and suggest the importance of using couple-level data.

